# God's mind on morality

**DOI:** 10.1017/ehs.2021.1

**Published:** 2021-01-11

**Authors:** Rita Anne McNamara, Rebekah Senanayake, Aiyana K. Willard, Joseph Henrich

**Affiliations:** 1 Centre for Applied Cross-Cultural Research, School of Psychology, Victoria University of Wellington, Wellington, New Zealand; 2 Centre for Culture and Evolution, Division of Psychology, Brunel University London, London, UK; 3 Department of Human Evolutionary Biology, Harvard University, Cambridge, MA, USA

**Keywords:** Moral reasoning, supernatural agent belief, culture and cognition, culture cognition co-evolution, cultural evolution, social cognition, cognitive anthropology

## Abstract

Most research on cognition behind religious belief assumes that understanding of other minds is culturally uniform and follows the Western model of mind, which posits that (a) others’ thoughts can be known and (b) action is best explained by mental state inference. This is potentially problematic if, as a growing body of evidence suggests, other populations view minds differently. We recruit Indigenous iTaukei Fijians who hold (a) a model of mind that discourages mental state inference and (b) co-existing Christian (Western) and traditional supernatural agent beliefs. Study 1 (*N =* 108), uses free-listing to examine how Western and local models of mind relate to beliefs. The Christian God cares about internal states and traits (aligning with the Western model of mind). Study 2 tests whether evoking God triggers intent focus in moral reasoning. Instead, God appears to enforce cultural models of mind in iTaukei (*N =* 151) and North Americans (*N =* 561). Expected divine judgement mirrors human judgement; iTaukei (*N =* 90) expect God to emphasise outcome, while Indo-Fijians (*N =* 219) and North Americans (*N =* 412) expect God to emphasise intent. When reminded to think about thoughts, iTaukei (*N =* 72) expect God to judge outcomes less harshly. Results suggest cultural/cognitive co-evolution: introduced cultural forms can spread new cognitive approaches, while Indigenous beliefs can persist as a reflection of local institutions.

## Graphical abstract

**Figure:**
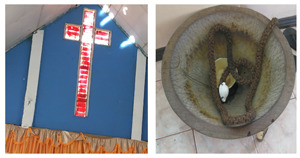

**Media summary:** Western belief's global spread is feared to homogenise cognition, but new belief may instead be co-opted by old patterns.

## Introduction

Through colonialisation and globalisation, Western cultural norms and institutions have spread to many societies across the globe. This has produced a level of cultural homogenisation that has long led researchers to wonder whether this cultural homogenisation would also result in cognitive homogenisation (Cox, 2000; Graves & Graves, 1978; Rozin, 2010). While some work shows individualism spreading and older generations looking more culturally distinct than their younger counterparts (Rozin, 2003; Santos et al., 2017), other evidence suggests a more continued blending of cultural knowledge sets and ways of thinking among immigrants (Reid, 1990; Vandebroek & Balick, 2012). Examination of the elements of cognition that remain from a heritage cultural worldview or are modified by intercultural contact may therefore provide a window into the dynamics of cultural evolution as it occurs within human cognitive systems.

A cultural-cognitive co-evolutionary process posits that, as components of culture at the ideological/belief level shift, they further imply shifts in norms and rules that structure social life, which in turn flow on to influence the cognitive processes used to interpret and respond to a given social situation. We focus here on religious belief; religious beliefs interlace with and influence different normative rules structuring social interactions. These rules in turn re-configure the attentional and motivational affordances that cognitive systems can work upon to enable individuals to navigate and thrive in particular social contexts. Some religious traditions have spread around the globe, yet syncretic elements of local beliefs are often retained (Grayson, 1992; Watanabe, 1990). Part of the variation across these beliefs includes variation in what supernatural agents are believed to care about, see and do (Norenzayan et al., 2016; Purzycki, 2013). An evolutionary and cognitive science of religion account of this variation would suggest that these beliefs vary partly as a function of the ecological systems believer groups live in, and partly as a function of pan-human social cognitive mechanisms (J. L. Barrett, 2011; Boyer, 2001; McNamara & Purzycki, 2020). We explore the cultural evolutionary influences on belief as two distinct religious traditions intersect with two distinct ways of approaching other human minds within a group of believers who live in a rapidly changing cultural environment.

### Evolutionary and cognitive science approaches to religion

Where does the human ability to conceive of and perceive of the divine come from? One strong set of answers comes from looking at how human minds conceptualise the mind(s) of god(s) as an extension of the ways we evolved to understand other human minds (J. L. Barrett, 2011; Boyer, 2001; Guthrie, 1995). Cognitive science of religion places human cognitive systems at the root of supernatural beliefs, with theorists further suggesting that this human-perception cognitive system either co-evolved with or was co-opted/exapted into further functional use as a means of bolstering human cooperation (Johnson, 2009; Norenzayan et al., 2016; Schloss & Murray, 2011; Sosis, 2009).

The cognitive systems theorised to be involved in supernatural agent perception are centralised on the social-cognitive processes that enable mentalising and theory of mind (Chudek et al., 2017; W. M. Gervais, 2013). These include the perception of human-like characteristics in non-human objects or entities (anthropomorphism, e.g. Guthrie, 1995) and potential biases towards giving objects/entities agency under certain motivational conditions like social isolation or stress (Waytz et al., 2010). Correlational data indicate that theory of mind differences in neuro-atypical populations can lead to lower levels of individual belief in supernatural agency (Norenzayan et al., 2012) and more fine-grained differences in facets of mentalising tendencies can further distinguish religious vs. spiritual believers (Genovese, 2005; Willard & Norenzayan, 2017). Brain imaging studies also suggest that believers infer the mind of God by imputing their own mind in the place of God's mind in order to answer questions about what God might think (Epley et al., 2009). These data taken together provide a strong foundation of theory that religious cognition starts as social cognition.

### Cultural models of mind

Despite these strides to map out a cognitive foundation of religious experience, the existing work remains constrained by a limited view of culture; much of the existing evolutionary and cognitive science of religion assumes a Western model of the mind (Lillard, 1998; Luhrmann, 2011). In the Western model, the mind (housed in the brain) is the origin point of all action. Individuals operate more or less autonomously, driven by inferred phenomena like preferences, instincts and goals that originate in the mind. This Western model of mind posits that knowing another mind is the same as knowing another being, with the ability to infer the contents of another mind theorised to be an essential cognitive requirement for survival as a social species (D'Andrade, 1987; Heyes & Frith, 2014; Malle, 2006; Povinelli & Giambrone, 2001).

Research from Western cultural traditions, and psychology in particular, perpetuates this view that perceiving the mind as the epicentre of all human action is a human universal (see Lillard 1998). However, other cultural approaches to the problem of understanding other people do not place the individual mind on such a pedestal. In this paper, we focus on a cultural approach to understanding others that starts from the premise that the minds of others are unknowable. This set of norms, known as the Opacity Doctrine or Opacity of Mind, suggests that the mind is contained within the opaque vessel of the head. We can never truly know what another person is thinking. The mental workings of another are private, and it is rude to intrude into that private mental space to make open inferences about anything beyond what the person themselves overtly states (Duranti, 2015; Groark, 2008; Throop, 2012).

Much of the existing cross-cultural work on mental state reasoning remains bound to the Western model of mind owing to the methods and theories used to measure it being produced predominantly by and for researchers from WEIRD (Western, Educated, Industrialised, Rich, Democratic: Henrich et al., 2010) societies, and therefore remains biased against finding the deeper nuances of how people in non-WEIRD societies are navigating social situations. Despite these limitations, previous work within communities that adhere to Opacity of Mind norms suggests that children in these communities still pass false belief tasks (albeit at older ages: H. C. Barrett et al., 2013; Callaghan et al., 2005). These studies have shown evidence for both uniformity and variation in the onset and development of the mental state reasoning abilities needed to pass tasks such as false belief (Callaghan et al., 2011; Dixson et al., 2017; Mayer & Trauble, 2012; Shahaeian et al., 2011; Taumoepeau, 2015). Further, there appears to be less variability in development shown across implicit rather than explicit theory of mind measures (with the caveat that implicit theory of mind measures are themselves subject to ongoing conceptual clarification, see Heyes, 2014; Kulke et al., 2018, 2019; Low & Perner, 2012; Overwalle & Vandekerckhove, 2013).

Among adults, Opacity of Mind norms appear to shift emphasis in moral reasoning towards outcomes rather than intentions (H. C. Barrett et al., 2016; Curtin et al., 2020; McNamara et al., 2019). For example, many with a Western model of mind would focus on intent, leading to judgements that an attempted murder (a bad intention with a good outcome) is worse and more worthy of more punishment than an accident (a good intention with a bad outcome). This is because the source of the violation in this case is the thought, and bad thoughts are in-and-of-themselves problematic. On the other hand, the outcome-focused judgement associated with Opacity of Mind norms would find the bad outcome of the accident worse and more worthy of punishment; the source of the violation in this case is the action, which is deemed more important than the intent. The data in these studies show that the people in these Opacity of Mind contexts use intent in judging these actions, which suggests that intent reasoning is to some degree of universal. However, these mechanisms might be developed under different conditions and function towards slightly different social-cognitive ends in the Opacity of Mind context.

### The minds of god(s) when human minds are opaque

If humans do indeed conceive and perceive the minds of gods as a function of their perception of human minds, then examinations of religious beliefs can provide a window into the changes that may occur in social cognition as cultural traditions are adopted by new groups and merge with existing beliefs. Working from a cultural evolutionary psychological framework, we may hypothesise that religious traditions will carry the social cognitive marks of the model of mind present within the culture where that religious tradition was developed. Further, as religions spread through various forms of intercultural contact, the social cognitive signatures of their originating cultural model of mind may be adopted along with the new religious belief sets.

Among Abrahamic traditions, Protestant Christianity stands out as one of the most mind-focused religious belief sets. Unlike Jewish or Roman Catholic participants, Protestants were more likely to say that a mere thought of wrongdoing is sufficient to constitute sin (Cohen & Hill, 2007; Henrich, 2020). Protestantism itself is highly intertwined with the advent of the Western sense of individualised and bounded self at the centre of the Western model of mind (Taylor, 2007). The shift away from ritual practice to individualised belief within the Protestant Reformation further pushed the mark of salvation inward, emphasising the importance of belief (Laine, 2014). Missionisation has led to the increase of Christianity in general, and Protestantism in particular, through many of the existing Opacity of Mind societies around the Pacific. Ethnographic research within these societies illustrates a level of distress that is caused by these introduced practices. As Protestantism encourages focus on mental states through emphasis on belief in God alone and the merit of intentions, it also places demands upon believers to speak openly and sincerely about their innermost states. Practices like confession were introduced and brought in novel open, public questioning and dialogue about one's internal world. This loss of the mental privacy and freedom that was previously found through mental opacity increased feelings of shame and previously uncommon behaviours like gossip (Duranti, 2008; Keane, 2008; Robbins, 2008; Robbins et al., 2014; Schieffelin, 2008). Though Fiji had a pre-contact history of ritualised practices like *i soro* (surrender), which are still used as means of repairing relationships in cases of wrongdoing, they are used on a broadly voluntary basis and do not go into the inner motivations of the wrongdoers as the confession practices in Christianity dictate (Arno, 1976). As detailed below, the introduction of various Protestant denominations has introduced points of tension in communities as they seek to balance traditional and introduced means of interaction.

### Ethnographic sketch of Fiji

Fiji is an interesting test case to explore cognitive impacts of cultural evolution through introduced religions. Pre-contact Fijian society revolved around kin-based hierarchies that concentrated power in hereditary *turaga* chiefs. These chiefly powers were bolstered by a *bete* priestly class in charge of managing connection with the ancestral spirits, or *Kalou-vu* (literally. ‘root god’). *Kalou-vu* are typically said to have founded communities as a set of five brothers who determine their descendants’ roles and obligations based upon which brother they descend from (Hocart, 1912; McNamara & Henrich, 2017). While Wesleyan Methodist Christianity is often considered a foundational part of personal Indigenous iTaukei Fijian identity (Purzycki et al., 2018), many practices and beliefs retain syncretic elements with traditional beliefs, particularly within the domains of traditional medicine (Katz, 1999), chiefliness (M. M. Gervais & Fessler, 2016) and connections to ancestral lands (M. M. Gervais & Fessler, 2016; Toren, 2004). Newer introductions of Pentecostal beliefs have further disrupted this balance among traditional and introduced Christian beliefs by re-branding *Kalou-vu* as *tevoro* (devils) and actively treating traditional practices as witchcraft (Newland, 2004). Local traditions of hospitality, kava drinking and food sharing are further contradicted by Pentecostal beliefs, which often exacerbates the tensions that arise from a shifting sense of social obligation from these introduced beliefs (Brison, 2007).

### Indo-Fijians

Fiji is also home to Indo-Fijians, originally brought from India to Fiji by the British between 1879 and 1920 to work on sugar cane plantations as indentured labourers (Gillion, 1962). The Indo-Fijians work largely as wage labourers and farmers on the larger Islands of Fiji. Although this population does not have Opacity of Mind norms, they may also hold a model of mind that is somewhat different from the Western one, differentiating mental states directed towards others from those relevant to the self (Willard & McNamara, 2019). This may relate to findings from India suggesting that traits related to impression management were rated more highly in India than in a German sample (Sharma et al., 2009).

### Overview of studies

As the review of the ethnographic literature above shows, there are many behavioural changes that happen when communities with Opacity of Mind norms adopt the mind-focused beliefs and practices of Christianity. Does this cultural introduction of Christianity also introduce a Western way of thinking about minds into an Opacity society? We find that, to the contrary, God concepts appear to bolster the locally salient models of mind in all three of our study populations.

## Study 1: free listing interviews

We first examine how Indigenous iTaukei Fijian participants’ concepts of Christian God vs. Local *Kalou-vu* ancestor spirits are associated with concern about humans’ internal states or external behaviours. We predict that, because the Christian God is associated with a cultural tradition of mind-focus, the Christian God will be more associated with concerns about internal states. Similarly, the Local *Kalou-vu* ancestor spirits will show the opaque-mind cultural tradition they arise from with more associations with external behaviours. We also examine whether supernatural agents care sufficiently about internal states vs. external behaviours to actively punish particular domains of wrongdoing. We again predict that the Christian God will be more associated with internal states while local *Kalou-vu* ancestor spirits will be associated with external behaviours.

### Method

#### Participants

We recruited 108 iTaukei Fijians (57 women; ages 18–75, mean 38.38; years of formal education 3–15, mean 9.68) in June 2013.

#### Materials

Study materials were translated into Standard Fijian and back-translated into English by two research assistants fluent in both languages. Free-listing questions focused on three agents: (a) the Christian God; (b) local *Kalou-vu* ancestor spirits; and (c) Police (acting as a human control for our two supernatural agent targets). See Supplementary Information for question details. All free-list data were translated into English and subsequently compiled and coded by McNamara. All original open-ended Fijian and English responses, along with coded data and other study materials, are available on the study's OSF project page (https://osf.io/mzky8/?view_only=5106f2dbcbfb4915bdca6ab83e7c625b). Items were coded first by eliminating variations that did not convey extra meaning (correcting spelling errors, transforming variations of the same word into the same root as in, for example, conjugated verbs, ‘helped’ and ‘helping’ becoming ‘help’). These were then aggregated from more specific instances into broader categories, including items like paired positive and negative synonyms (e.g. telling the truth and not telling lies both coded as honesty) and specific actions that could fall into a larger domain (e.g. sharing food and helping a neighbour both coded under the broader category of cooperation).

#### Procedure

Participants were interviewed in their homes or a neighbouring house in their village according to their availability by an iTaukei Fijian research assistant fluent in both Standard Fijian and English. Interviews lasted approximately 60 minutes and were administered in a single setting, along with interview questions for other simultaneously running projects (Purzycki et al., 2016, 2018).

### Results

We analysed free-list data using the AnthroTools package (Purzycki & Jamieson-Lane, 2016) for R (Team, 2008; see Supplementary Information for further details on the mathematics behind this technique). Table 1 shows items listed for each agent (Christian God = BG, *Kalou-vu* (local ancestor sprits) = KV, Police = PO) with salience scores of ≥0.09. Mean salience indicates how salient the item was on average for those who listed it, while Smith's *S* gives an indication of how salient the item was across the sample. Across these scores, we can get an idea of how common the item is across the concept and, if it is less commonly listed, how top-of-mind that item is for those who do include it.

**Table 1.** Sample salience scores ≥0.09 for what agents like and do not like (*N* = 105). Mean salience is the average individual item salience among individuals who listed the item, Smith's *S* is the salience of the item across sample and *n* is the number of participants who listed the item

		Item domain	Mean salience	Smith's *S*	*n*		Item domain	Mean salience	Smith's *S*	*n*
Christian God (BG)	Likes					Dislikes				
		Prosocial	0.75	0.52	72		Anti-social	0.76	0.55	76
		Obedience	0.84	0.40	50		Disobedience	0.75	0.33	46
		Ritual	0.84	0.13	16		Anti-Christian	0.75	0.22	31
		Honesty	0.66	0.12	20		Dishonesty	0.80	0.17	22
		Moral purity	0.69	0.11	16		Failed Cooperation	0.70	0.14	21
*Kalou-vu* (KV)	Likes					Dislikes				
		Ritual/substance	0.95	0.70	78		Christian	0.90	0.73	86
		Ritual	0.69	0.17	26		Vanua	0.79	0.19	25
		Anti-Christian	0.68	0.12	19		Ritual/Christian	0.85	0.13	16
		Vanua	0.90	0.09	10					
Police (PO)	Likes					Dislikes				
		Law abiding	0.71	0.54	80		Law breaker	0.70	0.53	79
		Obedience	0.78	0.51	69		Anti-social	0.59	0.46	81
		Cooperation	0.61	0.45	77		Failed Cooperation	0.70	0.44	65
		Prosocial	0.56	0.42	78		Violence	0.56	0.32	60
		Honesty	0.68	0.31	48		Dishonesty	0.70	0.31	47
		Anti-violence	0.59	0.09	16		Disobedience	0.82	0.28	36
	How punished									
Christian God (BG)		Physical harm	0.95	0.82	90					
		Natural disaster	0.69	0.44	66					
		Misfortune	0.53	0.16	32					
	How punished					What punished				
*Kalou-vu* (KV)		Physical harm	0.97	0.95	102		Vanua	1.00	1.00	50
		Misfortune/Vanua	0.55	0.25	47		Improper ritual	0.50	0.02	2
		Misfortune	0.62	0.14	24					
		Possession	0.49	0.14	29					

As we expect the Christian God to be more associated with inner states and traits, we expect more items that relate to internal qualities like morality, while the *Kalou-vu* remain more focused on actions like ritual. As with previous findings about the distinctions between more moralistic, universalising Gods like the Christian God, we similarly expect the Christian God to be associated with wide-scale impacts like climactic events, while the *Kalou-vu* remain focused primarily on local affairs.

Christian God and Police share similar likes/concerns and dislikes including obedience and cooperation; both are highly interested in honest behaviour. The *Kalou-vu*, on the other hand, care almost exclusively about ritual behaviour like *gunu yaqona*, ‘drinking kava’, and *drodroti*, ‘worshiping spirits’, anti-Christian acts and traditional issues like respecting the Vanua. *Kalou-vu* dislike both Christianity and disrespect for traditional norms. While these *Kalou-vu* concerns do not fall within the scope of what would often be considered universalistic moral norms, the kind of prosociality they may encourage is more directly focused at the local community level, as has been shown in previous work (McNamara & Henrich, 2017)

Both Christian God and local ancestor spirits punish with physical harm (e.g. *tauvimate*, ‘illness’, and *mate*, ‘death’) and misfortune (e.g. *sovatia na dredre/dredre na bula*, ‘difficulties in life’). The Christian God is unique in being able to punish with natural disasters like *cagilaba*, ‘cyclones’, and *dausiga*, ‘drought’, while the local ancestor spirits can punish with *maduataki*, ‘being shamed (in the village)’, and *curumi tevoro*, ‘spirit/devil possession’. Importantly, although the *Kalou-vu* generally dislike Christian activities, the majority of what they punish are things to do with violating village/vanua norms (e.g. *kosakosa/mamaue*, ‘unwanted noise’, and *beka turaga/beka koro*, ‘disrespecting the chief/village’).

We also examine the word usage for each of the target agents to see if there might be a difference in the agent's focus reflected in the ways that participants talk about them. In particular, the words *yalo*, ‘spirit’, and *dau* (a particle indicating actions indicating traits, e.g. *dauqoli* = fisherman, or person who fishes/a fisherman, *dauvinaka* = a person who is good because they are habitually seen being good) might be used more when the agent is thought of as having more of a focus on the internal characteristics and traits of a person rather than their behaviours. We find that around 25% of items listed for the Christian God use either word (*yalo*, 13%; *dau*, 12%), while these words are used in only 2% of listed items for *Kalou-vu* items (*yalo*, 1%; *dau*, 1%; 20.60× less likely than BG; odds ratio (OR) = 20.60, CI.95 [10.96, 43.34]) and only 4% of Police items (*yalo*, 1%; *dau*, 3%; 9.73× less likely than BG; OR = 9.73; CI.95 [6.73, 14.35]).

## Study 2: moral violation vignettes – manipulate intent and outcome

With the above evidence that the Christian and local traditional spiritual beliefs do indeed show markers of different cultural models of mind, do these Christian mind-focused beliefs have further cognitive downstream effects to increase mind focus in moral reasoning? We explore how mentalising is applied in socio-moral reasoning because this allows us to examine mentalising without directly asking participants in Opacity of Mind communities to state their (potentially rude/gossiping) inferences about another's state of mind (H. C. Barrett et al., 2016; Curtin et al., 2020; McNamara et al., 2019).

Study 2 uses moral violation vignettes that vary positive vs. negative intent and outcome, as shown in the intent/outcome matrix in Table 2 (McNamara et al., 2019; Young et al., 2011). This data was collected as part of a larger study; portions of this dataset were analysed in McNamara et al. (2019) to test a separate set of hypotheses. Our first analysis re-analyses the participants’ own judgements of the vignette characters that are reported in McNamara et al. (2019) as a function of whether they were asked about God or not. Our second and third analyses use new dependent variables: God's expected judgements of the vignette characters predicted as a function of perceived actor intent and victim outcome. These final two analyses differ from McNamara et al. (2019) in both the dependent variables used and in adopting a continuous measure of intent and outcome (rather than categorical from the vignette type) to add further nuance to how participants may expect judgements to be moderated by their own social perceptions.

**Table 2.** Intent/outcome matrix for intent conditions. Endorsements of stronger punishments against failed attempts indicate intent focus; stronger punishments of accidents indicate outcome focus

Outcome		Intent	
		Positive	Negative
	Positive	No violation	Failed attempt (e.g. attempted murder)
	Negative	Accident (e.g. Involuntary manslaughter)	Successful attempt/intentional violation (e.g. murder)

### Method

#### Participants

We examine evidence from three societies across four phases of data collection that ran from 2012 to 2014. Our first set of analyses examines data collected from 151 iTaukei Fijians (90 asked about God, 64 Not) and 561 North Americans (410 Asked about God, 151 Not). Our second set of analyses includes only those iTaukei Fijian and North American participants who were asked about God, and it adds 219 Indo-Fijians. Our third set of analyses focuses on data collected from 72 iTaukei Fijians from Yasawa Island in May–June 2014. See Supplementary Information for detailed sample descriptive statistics.

#### Judgment measures

We examine the average intent/outcome focus across domains (see supplement of McNamara et al., 2019, for detailed intent condition by domain by sample analysis). Materials were modified for all samples to reflect culturally appropriate names and moral violation content. Participants reported judgements of actions depicted in the vignettes for both what they think and what God thinks using a −2 (most negative/intentional/worthy of punishment) to +2 (most positive/accidental/worthy of reward) Likert scale, adapted from Barrett et. al. (2016). Judgments were always made in the same order: (1) Good/Bad, (2) Purpose/Accident, (3) Positive/Negative, (4) Pleased/Angered, (5) Other Opinion Good/Bad and (6) Reward/Punish.

#### Procedure

All participants followed the same basic procedure: they listened to or read a vignette, then answered questions about the vignette. This was repeated for four vignettes. Domains of moral violation (e.g. harm, theft, taboo) were crossed with intention conditions and counterbalanced across participants. Following each vignette, participants answered the six judgement questions followed by an open-ended question about what they thought of the violation to capture anything participants wanted to say that they felt they did not communicate through the judgement questions.

### Results

We carry out our analysis targeting three questions:

1. Does the mere mention of God evoke mentalising?
2. Do people predict that God will make the same judgements they do?
3. Do people's predictions of God's mind change with their own minds?

We build multilevel regression models for each question, each following maximal modelling (Barr et al., 2013) as applicable using lme4 (Bates, 2010) in R (Team, 2008). See Supplementary Information for full regression tables.

**Figure 1.**
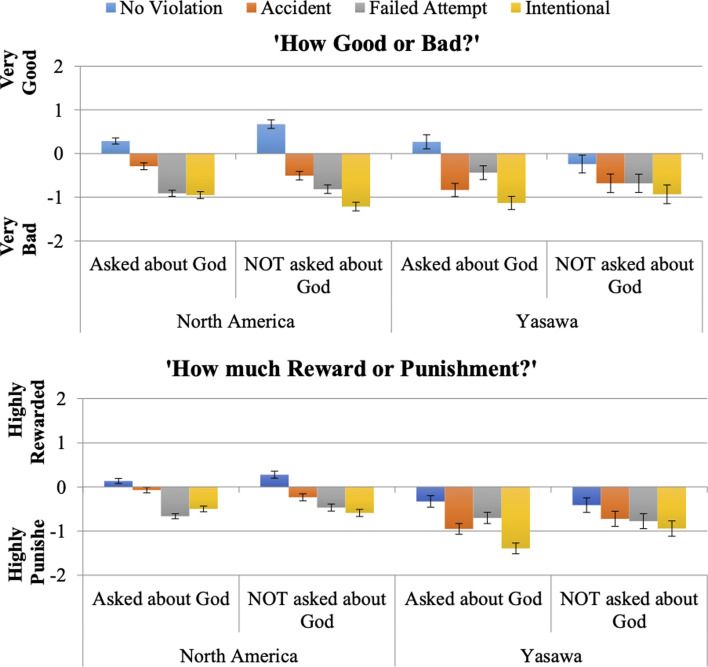
Cross-societal responses when asked vs. not asked about God while judging moral vignettes; asking about God enforces intent in North America and outcome in Yasawa (iTaukei Fijians).

To examine question (1), whether the mere mention of God acts as a sort of mentalising prime (Figure 1), we use the participant's *own* opinions of both how good or how bad the action was and how worthy of punishment or reward it was. We compare these across North American and iTaukei Fijian samples who were and were not asked about God.

We find that the two samples responded to intent conditions depending on whether they were asked about God for both Good/Bad (*F*(3, 5263) = 31.19, *p* < 0.001) and Reward/Punish (*F*(3, 5258) = 19.35, *p* < 0.001). We further decompose this interaction to compare accidents (positive intent with negative outcomes) and failed attempts (negative intent with positive outcomes). iTaukei Fijians who were asked about God report that accidents are worse (*b*_Accidents-Failed Attempts_ = 0.40, CI.95 [0.24, 0.55], *p* < 0.001) and more worthy of punishment (*b*_Accidents-Failed Attempts_ = 0.25, CI.95 [0.12, 0.37], *p* < 0.001). However, this difference disappears when Fijians are *not* asked about God (Good/Bad *b*_Accidents-Failed Attempts_ = −0.001, CI.95 [−0.22, 0.22], *p* =0.99; Reward/Punish *b*_Accidents-Failed Attempts_ = −0.05, CI.95 [−0.23, 0.12], *p* =0.56). So, our God prime does matter, but it seems to encourage a focus on outcomes over mental states.

North Americans who were asked about God judged failed attempts as worse than accidents (*b*_Accidents-Failed Attempts_ = −0.62, CI.95 [−0.72, −0.52], *p* < 0.001); more worthy of punishment than accidents (*b*_Accidents-Failed Attempts_ = −0.59, CI.95 [−0.67, −0.51], *p* < 0.001); and more worthy of punishment than even intentional violations (*b*_Failed Attempts-Intentional_ = 0.16, CI.95 [0.08, 0.25], *p* < 0.001). North Americans who were *not* asked about God also judged failed attempts as worse than accidents, but instead judged intentional violations as the worst overall (Good/Bad *b*_Failed Attempts-Intentional_ = −0.40, CI.95 [−0.51, −0.29], *p* < 0.001; Reward/Punish *b*_Failed Attempts-Intentional_ = −0.12, CI.95 [−0.21, −0.03], *p* = 0.008).

**Figure 2.**
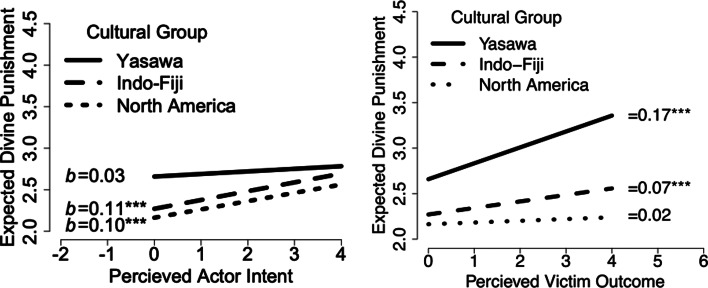
Expected divine punishment by society based on (a) actor intent and (b) victim outcome. Yasawans expect God to punish bad outcomes more than bad intentions, in line with their personal judgements (see McNamara et al. 2019).

To examine question 2, our second analysis uses participant's ratings of *what they think God thinks*, in this case how much punishment to direct towards perpetrators, as a function of perceived intent and outcome (Figure 2). Divine judgement across North American, iTaukei Fijian and Indo-Fijian samples tracked along with each society's normative stance on focus towards intent vs. outcome in human judgements (McNamara et al., 2019). For both North American and Indo-Fijian participants, increased actor intent predicted a similar increase in divine punishment (*b*_North America_ = 0.10, CI.95 [0.07, 0.13], *p* < 0.001; *b*_Indo-Fiji_= 0.11, CI.95 [0.07, 0.14], *p* < 0.001), while changes in actor intent did not predict any change among iTaukei Fijian participants (*b*_Yasawa_= 0.03, CI.95 [−0.05, 0.11], *p* = 0.44). On the other hand, iTaukei participants did expect significantly more divine punishment for more negative outcomes (*b*_Yasawa_= 0.17, CI.95 [0.08, 0.27], *p* < 0.001). North American participants did not expect divine punishment to increase with negative outcomes (*b*_North America_ = 0.01, CI.95 [−0.03, 0.06], *p* = 0.42), although Indo-Fijian participants did (*b*_Indo-Fiji_= 0.07, CI.95 [0.03, 0.11], *p* = 0.001).

For our final set of analyses, we examine whether contextual factors that shape human judgements lead participants to expect that God will respond in a way similar to or different from humans (question 3). We analyse a subset of data in which iTaukei Fijians were primed with thinking about thoughts vs. thinking about actions. Participants were primed with questions asking to list things that supernatural agents would punish or reward people for. These targets of punishment or reward could be actions (the Action prime) or thoughts (the Thought prime). Following these primes, the procedure continued to the same method of presenting of moral norm vignettes followed by judgement questions as used in our previous two analyses (see Supplementary Information and McNamara et al., 2019 for further procedure detail). In this analysis, we again focus on what participants think God will think.

**Figure 3.**
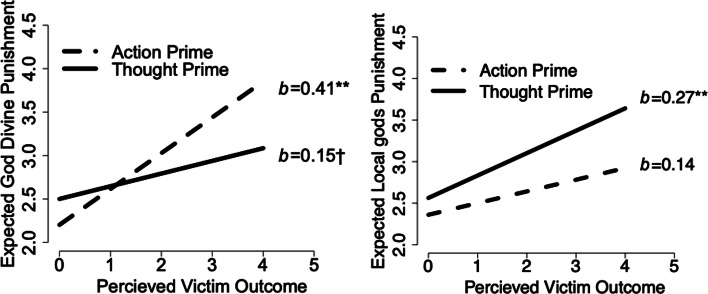
Expected divine punishment by action or thought prime based victim outcome for those reminded of the Christian God (a) and *Kalou-vu* (b). The thought/action prime had a significant effect when asked about Christian God, but not *Kalou-vu*. God is expected to punish bad outcomes significantly more when reminded of actions but not thoughts; *Kalou-vu* show the opposite effect.

We run two models: one on the answers iTaukei Fijian participants gave for primes asking about the Christian God, and a second on the answers these same participants gave for primes asking about the *Kalou-vu*. We look for patterns of answers as a function of the primes, perceived intent and outcome with an interaction between prime (two-level categorical), perceived actor intent (continuous) and perceived outcome (continuous; see Figure 3).

When asked about the Christian God, participants on average expected more divine punishment following the Action prime (*b*_Action-Thought Prime_= 0.30, CI.95 [0.05, 0.55], *p* = 0.02), which showed a different pattern of effects based on outcome (*F*(1,127) = 9.55, *p* = 0.002). Our prime may also have increased the divine punishment from the *Kalou-vu*, although the effect is not well estimated (*b*_Action-Thought Prime_= 0.20, CI.95 [−0.12, 0.53], *p* = 0.22).

We decompose interactions between primes and perceived outcome vs. intent. For those primed with questions about the Christian God, perceived actor intent failed to significantly predict expected divine punishment regardless of prime (slope of perceived intent in Action prime, *b* = 0.02, CI.95 [−0.10, 0.25], *p* = 0.72; slope of perceived intent in Thought prime, *b* = −0.01, CI.95 [−0.14, 0.11], *p* = 0.79).

However, more severe outcomes predicted greater divine punishment when primed with Actions (slope of perceived outcome in Action prime, *b* = 0.41, CI.95 [0.28, 0.54], *p* < 0.001; slope of perceived outcome in Thought prime, *b* = 0.14, CI.95 [0.01, 0.28], *p* = 0.053). This difference between expected divine punishment was statistically significant (*b*_Action-Thought Prime_= −0.27, CI.95 [−0.44, −0.10], *p* = 0.002).

While the primes had only weaker or poorly estimated effects for those reminded of *Kalou-vu*, expected divine punishment does trend higher for more severe outcomes when primed with Thoughts (slope of perceived outcome in Action prime, *b* = 0.14, CI.95 [−0.05, 0.33], *p* = 0.17; slope of perceived outcome in Thought prime, *b* = 0.27, CI.95 [0.10, 0.45], *p* = 0.03). This difference, *b*_Action-Thought Prime_= 0.13, CI.95 [−0.09, 0.35], is suggestive, although not significant at conventional cut-offs (*p* = 0.28). Similar patterns emerge for intent, although the Thought prime is measured with more precision; the slope of perceived intent in Action prime is *b* = 0.16 (CI.95 [−0.03, 0.35], *p* = 0.12) and that of the perceived intent under the Thought prime is *b* = 0.18 (CI.95 [0.01, 0.34], *p* = 0.05).

## Discussion

Although Christian beliefs convey a Western model of mind among our Indigenous iTaukei Fijian participants (Study 1), we find that they maintain the local, outcome-focused model when activated in moral reasoning, by way of divine minds being expected to judge similarly to human minds (Study 2). In Study 1, we find both the Christian God and Police are believed to share similar concerns and dislikes, supporting the theory that supernatural agents of moralising prosocial religions hold a similar function to secular police (Kay et al., 2010; Norris & Inglehart, 2004). *Kalou-vu*, on the other hand, are restricted to caring about rituals and local concerns of the *vanua* (local land and its people) – specifically, whether people are acting with appropriate respect for the local norms of the community. *Kalou-vu* are widely considered to be antithetical to Christianity, reflecting some tension between traditional and Christian beliefs (Newland, 2004; Tomlinson, 2004). We further find that the *Kalou-vu* care more about ritual and local village respect, reflecting both the different focus of the tradition the beliefs come from and some of the local orientation of their potential prosocial effects (Katz, 1999; McNamara & Henrich, 2017).

Word usage in the listed items also suggests that the Christian God cares more about mental states and characteristics than the other two agents. Despite the overall similarity in domains of concern between the Christian God and Police, the Christian God appears to care more about the unseen elements of human actors’ internal states (shown by the higher use of words like *yalo*, ‘spirit’, and *dau*). Thus, we see some evidence for the God concept among our participants having the components of a maximally effective third-party punisher capable of supernatural agent policing (Norenzayan et al., 2016) and the Western model of mind embedded in Protestant Christian tradition (Cohen & Hill, 2007; Taylor, 2007).

Despite this conceptual view of God, reminders of the Christian God do not themselves provoke a focus on mind within those who hold these beliefs; they do not promote intent focus in the Opacity of Mind context in Fiji. Across North American, Indigenous iTaukei Fijian and Indo-Fijian participants, God's expected judgements, and whether these focus more on an agent's intent or the outcome of their actions, mirror people's own judgements (McNamara et al., 2019). This suggests that people in communities with different models of mind may infer the mind of God as mirroring the minds of humans within that context, similar to results found in Western societies (Epley et al., 2009; Schjoedt et al., 2009). When iTaukei are reminded to think about thoughts, they expect God's punishment to be less severe for bad outcomes than when reminded of actions, similar to shifts in their own judgements that become less outcome focused when primed to think about thoughts. This effect emerges when primed to think about the Christian God, but not for *Kalou-vu*. This may also reflect the different socio-cultural functions and cultural-evolutionary histories of these sets of beliefs (McNamara & Henrich, 2017; Willard et al., 2020).

When looking across the results from our cross-site comparisons, it appears that our iTaukei Fijian participants might be responding with higher punishment ratings overall. We would discourage this interpretation as there may be some differences in how participants in each society used the scales, which will limit our ability to make these direct cross-site comparisons (Fischer & Poortinga, 2018). That said, might this pattern suggest the iTaukei conception of God is harsher than North Americans’ conception? One could interpret this as a reflection of the greater tightness in Fijian society that responds more harshly to any kind of norm violation (Gelfand et al., 2011). While we cannot definitively rule out this possibility, there are a few items in this data that suggest otherwise. First, our Indo-Fijian participants have similar cultural tightness, yet do not show the same degree of higher punishment expectations. Other studies with beliefs about Christian vs. ancestor spirits suggest that God is seen as far more benevolent than the *Kalou-vu* (McNamara, 2020). The idea of God as forgiving appears to have been retained in the package of concepts within Christian belief (McNamara & Henrich, 2017; Tomlinson, 2004).

We frame our results around the idea that culture (ideas, beliefs, values and practices) and cognition (how we think or processing information) can co-evolve (non-genetically). Such a cultural evolutionary process includes not only the ideas and beliefs that people transmit to each other, but also the norms and other rule-based structures that people develop within societies and structure daily life. These ideas and normative rules will further shape how people perceive, interpret and respond to the world around them at a cognitive processing level. This would happen at an attentional and motivational level, as the ideas and rules structuring life will make certain aspects more salient and dictate certain priorities for directing attentional resources. For example, in a world structured by situational rules that dictate behaviour like ties of kinship, the most important piece of information one can learn about another is how they are related. From this, introductory practices that emphasise family heritage and connections may develop as a normative procedure to facilitate individuals in orienting to each other upon first meeting (Fijians continue to observe such kinship-based introductions and naming practices). In these settings, the optimum strategy will favour attention to cues that indicate kinship status, making these elements both more readily brought to mind (salient) and more readily detected (sensitive). On the other hand, in a society with fewer situational elements like rules of kinship or other interpersonal obligations dictating action, the mental state of the actor is more informative because of the lack of other situational rules constraining action. Thus, one's preferences become a true indication of action, making behaviour a true window into the mind (the mind becomes knowable) and the best strategy for focus in determining how to interact with another agent (the mind becomes a focus). Thus, this simple socio-ecological difference between tighter norm-based societies like Fiji and looser more individualistic/autonomous societies like most of the Western world builds the foundation for different models of minds to emerge (Curtin et al., 2020; Henrich 2020). Ecologically, the association between stable environments and reliable governments has been correlated with increasing secularisation and increases in individualising, emancipative values, suggesting that the stability of the environment could be an overarching ecological pressure that underlies the normative social institutions that then filter down to shape the cognitive responses within individuals (Inglehart, 1997a, 1997b; Norris & Inglehart, 2004).

This cognitive adaptation through culture is likely to build upon evolved psychological mechanisms that are broadly available to most humans and modulated across development (Henrich, 2016; Laland, 2018). Linguistically, the introduction of novel words that can be used as tools to structure communication about minds has been shown to improve performance on classic theory of mind tasks (Pyers & Senghas, 2009). Our work expands this beyond language to the realm of beliefs and rituals, suggesting that religions that arise and co-develop within particular models of minds can carry these mental models with them. Previous work on outcome focus in the context of Opacity of Mind norms also suggests that intent focus forms a functional universal, in that it is present in all known societies but less readily brought to mind in some contexts (H. C. Barrett et al., 2016; McNamara et al., 2019). Within the scope of mentalising processes, this may further corroborate findings discussed in the introduction that suggest that implicit theory of mind is less impacted by culture than explicit theory of mind.

Our approach intersects with other research programmes. The idea that culture and cognition coevolve culturally has long been at the core of Dual Inheritance Theory (Henrich, 2008), and the specific notion that culture shapes our mentalising abilities has long been commonplace within anthropology (D'Andrade, 1987; Luhrmann, 2011). Broadly speaking, our approach is not inconsistent with recent formulations such as that of Heyes (2019).

We observe contents of belief by looking at the relative frequency of ideas in the participant population. Given the local context of Opacity of Mind norms, evidence for mind-focused norms, we infer, probably came from a different belief system. We further support this inference with our evidence that the association between internal states is higher for the introduced Christian beliefs than for the local *Kalou-vu* ancestor spirits.

After having shown that the idea of more internal mind focus is present in Fijian society via Christianity, we present Study 2 to show that these ideas have not made the leap into moral judgements. We show, instead, that God appears to uphold the local model of minds, rooted in Opacity of Mind norms. McNamara et al. (2019) propose different socio-ecological pathways which may have favoured the preservation of this local Opacity of Mind model, including the Cognitive Efficiency Hypothesis as suggested above (in highly norm-structured societies, minds are a poor predictor of behaviour and thus a poor target of focus) and the Relational Mobility Hypothesis, which more directly focuses on the special island ecology that peoples of the Pacific face. If one cannot get away from a neighbour in a conflict, then a novel normative approach that reduces focus on inscrutable intentions and shifts to observable actions could be favoured for group-level survival. Curtin and colleagues (2020) similarly posit kinship as a normative structure that could produce the environment favouring a less mind-focused approach to understanding others. All three imply that societies with less focus on individual autonomy may exhibit varying degrees of Opacity of Mind, with other aspects of mentalising like perspective taking perhaps being facilitated as the need to attend to subtle cues increases (Wu & Keysar, 2007).

Our findings provide further evidence for blending of cognitive forms as novel cultural forms expand across societies. This addresses several puzzles in both cognitive science and evolution of religion, as well as the cultural evolution of cognition.

Cognitive science of religion suggests that our ability to conceive of and perceive divine minds represents an outgrowth of our ability to perceive human minds. The data we show here partly supports this position, adding a layer of cultural nuance. The cultural histories of the belief systems themselves are also important to consider when projecting what aspects of human-focused social cognition might carry over into religious belief. Protestant Christianity may be among the most mind-focused religious belief systems owing to its history and deep roots within Western Europe and the mind-focused, increasingly individualistic cultural frame it entailed (Henrich, 2020; Laine, 2014). We show some preliminary evidence that this does not carry over to other religious traditions from very different cultural backgrounds.

However, this remains to be more fully fleshed out within a wider array of belief traditions and cultural models of mind. Other societies with Opacity of Mind norms like certain Mayan groups would be an interesting extension of this work, as they too have syncretic beliefs that unite Roman Catholic Christianity with Indigenous beliefs. Importantly, their Opacity of Mind context has a completely different cultural history to the Opacity norms found in Pacific societies like iTaukei Fijians (Luhrmann, 2011).

The work here remains a tantalising glimpse, and is ultimately preliminary. More work to gather fine-grained details about how cognition might or might not be carried along with cultural forms needs to be done with a far wider array of societies and practices. The best way forward to obtain the quality of data from wider demographic groups that still meets the rigour of replicable and open science is through broader collaboration networks. The present work is also limited in the time depth, as the processes we suggest require longer-term longitudinal work. Given the trajectory of Western cultural influence in places like Fiji through mechanisms like globalisation and international development, one might expect that the frequency of mind-focused approaches should increase over time. However, if the existing village norms persist, then the Opacity model should persist. Similarly, future studies should examine whether those who successfully adopt new models of mind are met with more success in domains like perceived prestige or reproductive success. At a group level, these beliefs may be found to promote long-term group survival as the ecological conditions shift to favour tighter or looser norm-structuring and lesser or greater focus on emancipative values (Gelfand et al., 2011; Inglehart, 1997a, 1997b; Norris & Inglehart, 2004). One may expect generational differences in cognitive processing associated with various beliefs in addition to the changes that may come along with exposure to novel cultural constructs. It remains to be seen how the developmental processes of enculturation as children and aging into adulthood may interact with the cultural cognitive variations that may come with exposure to novel cultural forms in traditional environments. The question further remains open as to how these traditional beliefs may transition into and persist in more urban environments. Some work in Fiji suggests that traditional kin networks remain active to support people in times of crisis (Campbell, 2014; Janif, 2014). These cultural solutions to urban problems may further carry cognitive signatures that stay with migrants as they move from rural to urban lifestyles.

This work hints at how cognition itself may evolve as a result of cultural transmission – or not. We see evidence of a novel, mind-focused model of mind in Christian God beliefs within this Fijian community. Nevertheless, our iTaukei Fijian participants maintain a local, outcome-focused approach when anticipating what God might think of human actions. This suggests that cognition might move along with new ideas, but those ideas themselves are modulated to the local socio-cultural adaptive pressures. As the world continues to become more interconnected through technology and education, one might expect a decrease in cognitive diversity to match (Rozin, 2010). However, our studies suggest a persistence of cognitive forms that remain relevant to the context. This may indicate a wider diversity of cognition than is currently captured in existing psychological data, as the tools we have to measure it rely upon the cultural view of what the researchers themselves believe to be the meaningful or appropriate way of even approaching a given cognitive task.

## Supplementary material

For supplementary material accompanying this paper visit https://doi.org/10.1017/ehs.2021.1. click here to view supplementary material
