# Supplementary material for: God's mind on morality
Source: Evol Hum Sci. 2021 Jan 11;3:e6. doi: 10.1017/ehs.2021.1 (PMC10427303; doi:10.1017/ehs.2021.1)
Supplement: Supplementary file 1 [file S2513843X21000013sup.zip › S2513843X21000013sup001.docx]

# Free Listing Analytical Technique

As described in (Purzycki et al., 2018), this method calculates the cognitive salience of items as a function of the primacy and frequency of the item across the sample’s lists, with calculations on the item rather than on the individual listing it. The key metric is the mean salience score, or Smith's S, which is calculated across the sample. Item salience (i) is calculated with Eq. (1):

$$i=\frac{n+1-k}{n}$$

where n is the total number of items an individual listed, and k is the order in which an item was listed. Smith's S (Eq. (2)) is the mean value of item across the sample (S. Borgatti & Halgin, 2011; Smith & Borgatti, 1997; Smith et al., 1995):

$$S= \frac{\sum i_{T}}{N}$$

In this equation, $i_{T}$ indicates item type and N denotes the total sample size. Smith's S will therefore increase as a function of how common the item is across lists and how early its occurrence is within lists. Smith's S values can sometimes be inflated due to repeated items; AnthroTools' “MAX” function was used to include only the earliest instance of any repeated items in the calculation.

# Free Listing Questions

For each of these agents, participants were asked to list up to 5 items in answer to:

• The kinds of things (agent) cares about or likes.

• The kinds of things (agent) dislikes.

A second set of free-listing questions focused on the two supernatural agents: 1) the Christian God and 2) Local *Kalou-vu* ancestor spirits. For each agent, participants were asked to list up to 5 things:

• What kinds of things does (agent) punish people for doing? (WHAT_Pun)

Participants were also asked to list up to 5 ways Local *Kalou-vu* ancestor spirits could punish them.

• How do the *kalou-vu* of this village punish people? (HOW_Pun)

# Free Listing Items Listed

Items listed for Christian God include specifics like *cakacaka/ daucakacaka* “hardworking” and *yalo vinaka/ yalo dina* “kindhearted” for Prosocial; *talairawarawa* “obedience” and *vakarorogo* “listening” for Obedience; *wilivola tabu* “bible reading” and *masumasu* “prayer” for Ritual; *dina* “true/ being truthful” for Honesty; and *savasava/ yalo savasava* “clean/ clean spirt” for Moral. Christian God dislikes *yalo ca* “bad spirit” and *vucesa* “laziness” for Anti-social; *talaidredre* “Disobedience;” *valavala ca* “sins/ bad acts” and not praying or reading the bible for Anti-Christian; *lasulasu* “lies” for Dishonesty; and *butako* “theft” for Failed Cooperation. Police like *muria na lawa* “respect the law” for Law Abiding; *talairawarawa* “obedience” and *vakarorogo* “listening” for Obedience; *kua na butako* “no theft” and *cakacakavata* “working together” for Cooperation; *loloma/ dauloloma/ vielolomni* “loving person” for Prosocial; and *dina* “true/ being truthful” for Honesty. Police dislike *basu lawa* “law breaker;” *viavialevu* “pretentious – lit. to want to be big” and *caca veiwekani* “bad family relationships/ bad relationships” (indicating everything from family troubles to gossip) for Anti-Social; *butako* “theft” for Failed Cooperation; *veivala* “fighting” for Violence; *lasulasu* “lies” and *lawaki ca* “deception; lit. evil deceiver who works by plotting” for Dishonesty; *talaidredre* “Disobedience;” and *vosavosa ca* “foul language” for Etiquette.

*Kalou-vu*, on the other hand, care almost exclusively about *gunu yaqona* “drinking kava” and *drodroti* “worshiping spirits” for Rituals; they also like *valavala ca* “sins” and *garogaro-ca* “lusts” for Anti-Christian; and *vakarokorokotaka na Vanua* “respect the land and it’s people” plus *vagagalu* “silence (in the village) for respecting the Vanua. Local Ancestor Spirits dislike *lotu dina* “true believers” and *vosa ni Kalou* “the word of God” plus going to church and praying for Christian; *vakosakosa* “excessive noise” and *sega ni rokovi na turaga/ beci na lewa ni Vanua* “not respecting the chief/ breaking the laws of the land and its people” for Vanua.


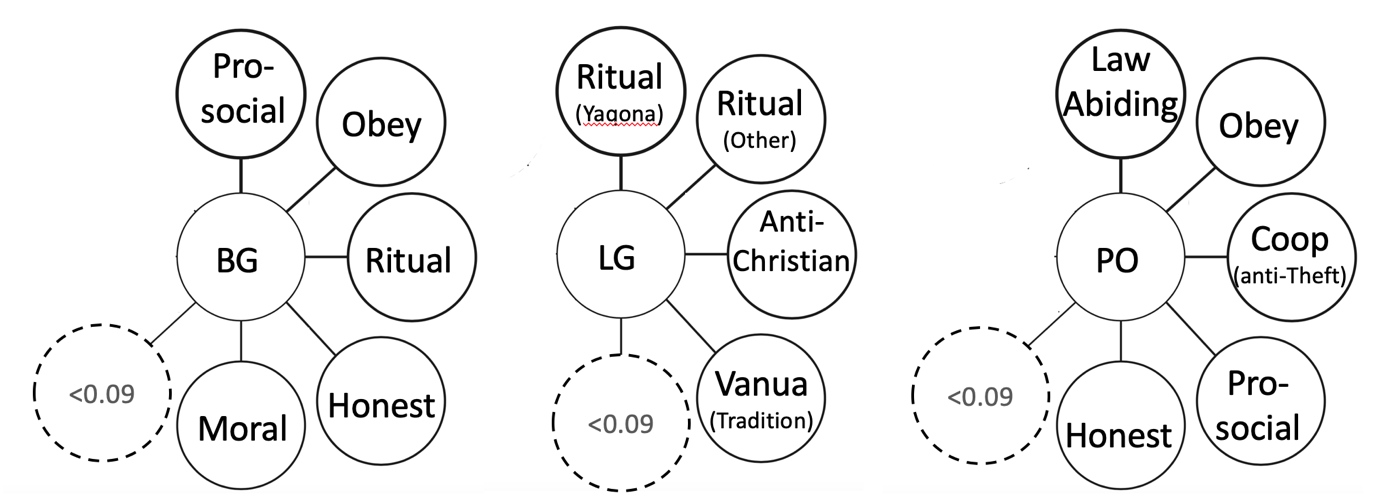


A Likes

B Dislikes


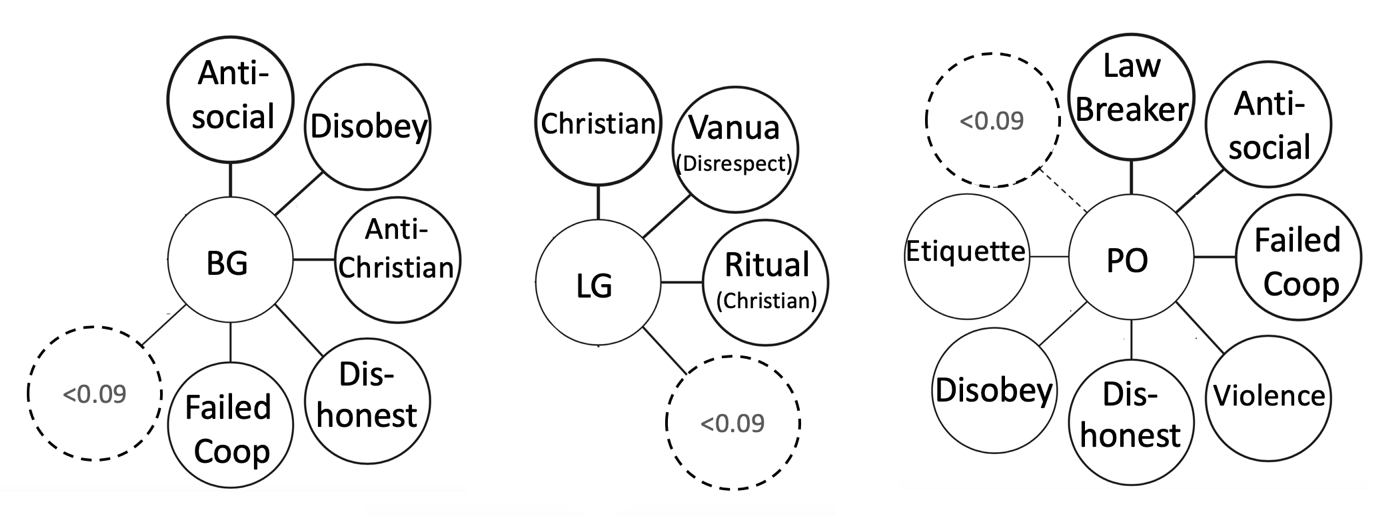


Figure 1 A Likes and B Dislikes items with Smith’s S >0.09. BG = Christian God; LG = *Kalou-vu*; PO = Police

A How Punish


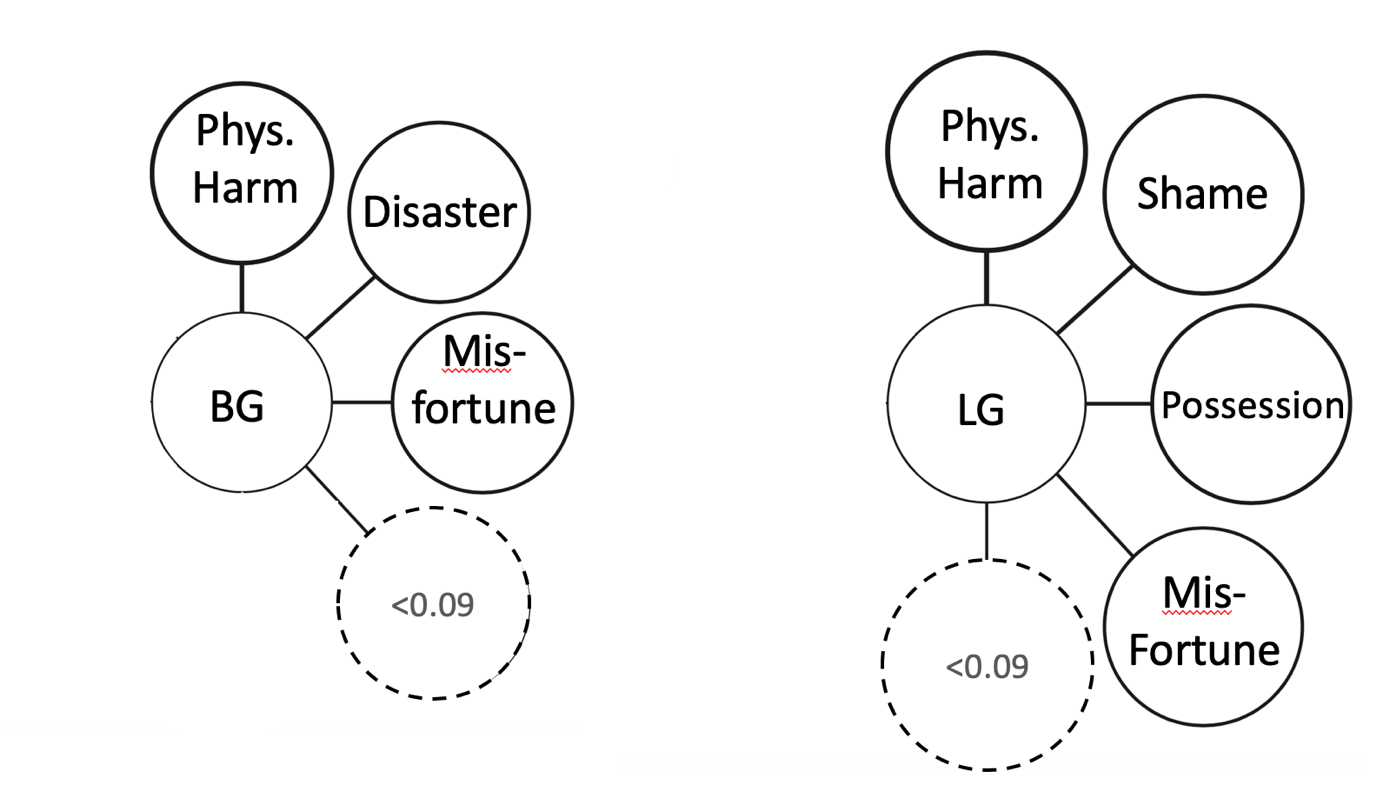


B LG Dislike vs. What Punish


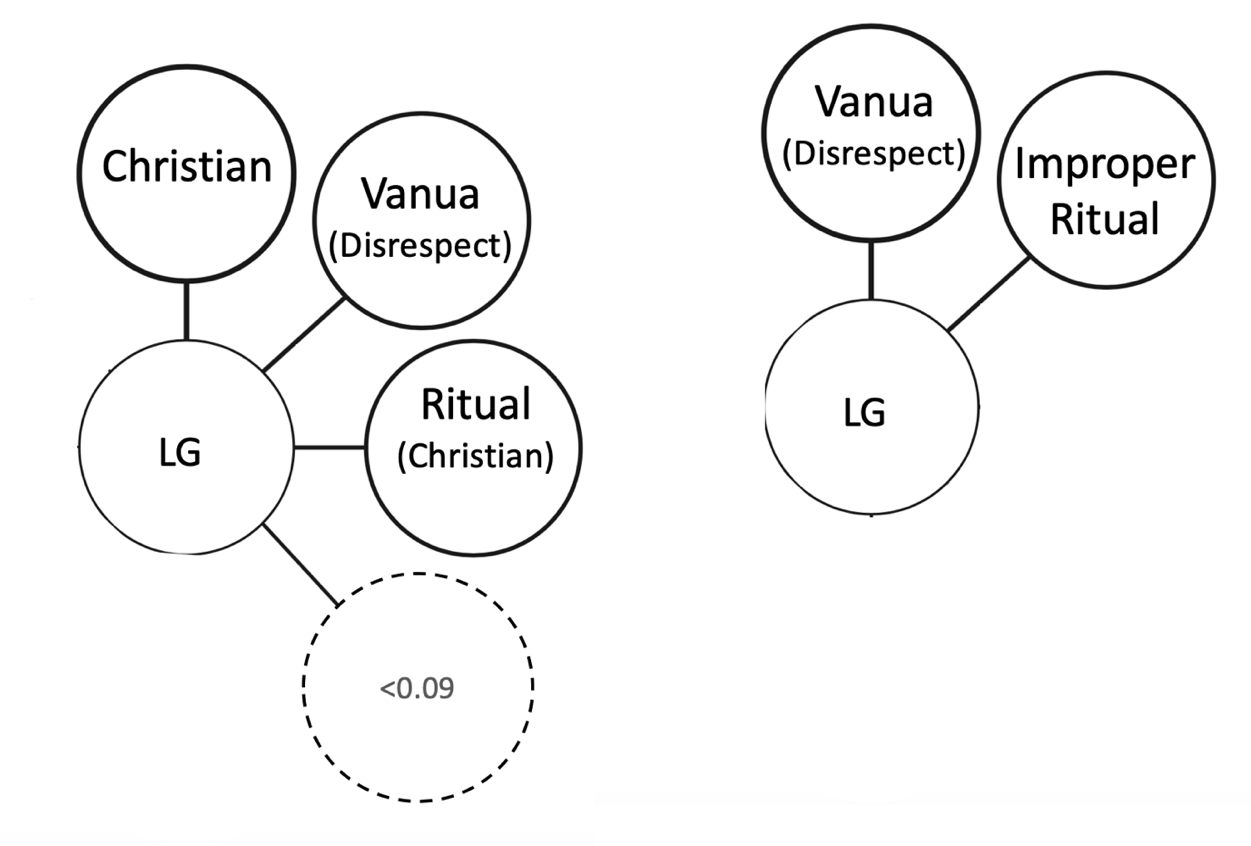


Figure 2 Items with Smith’s S >0.09 for A) How supernatural agents punish (BG = Christian God; LG = *Kalou-vu*) and B) Dislikes vs. What *Kalou-vu* actually punish.

# Study 2

Vignettes spanned six domains: (1) harm, (2) theft, (3) poisoning, (4) food taboos, (5) social taboos, and (6) failed cooperation.^[[1]](#footnote-1)^ We examine whether mere activation of Christian beliefs evoke mentalizing via more intent focus in iTaukei Fijians with Opacity of Mind norms and a comparison sample of North Americans without these norms. We then look at the patterns of moral judgments members of either society make as a function of whether or not Christian beliefs were activated at the time they were asked to make moral judgments. If Christian belief induces greater mind focus, we predict that our iTaukei Fijian participants will show more focus on intent by harsher judgments of failed attempts when reminded of the Christian God. This intent focus will be evinced by harsher punishments of failed attempts (e.g. attempted murder) relative to accidents (e.g. manslaughter) in cases when they are asked to think about God with questions about God. Conversely, when the questions about God are absent, this intent focus should again disappear and show the harsher treatment of accidents relative to failed attempts, as is in alignment with the Opacity of Mind behavioural/ outcome focus.

We then examine how this might be evident in humans’ expectations about what God might think. If reminders of mind-focused Christian beliefs do not induce more mind focus in human judgments, might we still see that the Christian God is still expected to care more about intention? Conversely, if the mind of God is indeed a reflection of the minds of believers, would God still be expected to care about intention or outcome in the same way as the believers themselves do? If the mind of God is a reflection of believers’ minds, then we predict that expectations of God’s divine punishment should mirror humans’ more outcome-focused judgment pattern in the Opacity of Mind context.

As a final test to examine how divine minds may reflect the minds of believers, we examine whether explicitly activating thoughts about thoughts (activating mentalizing) will also shift divine punishments toward more intent focus in an Opacity of Mind context. Reminders of thoughts have previously been shown to boost intent focus in human moral judgments within these Opacity of Mind communities (McNamara et al., 2019). We therefore predict that, if human believers are inferring the minds of supernatural agents as a reflection of their own minds, then activating the importance of thought as a target of judgment will similarly boost expectations of divine focus on intent through expectations of harsher punishment for intentional violations.

# Study 2 Sample Details

|  | Participants | Age | Education | Sex | Asked  about God | NOT Asked  about God |
| --- | --- | --- | --- | --- | --- | --- |
|  | N | Mean  (min.- max.) | Mean  (min. – max.) | N Women | N | N |
| Yasawa | 151 | 43.15  (18-80) | 9.33  (4-15) | 78 | 90 | 64 |
| US & Canada | 561 | 29.20  (17-72) | 13.99  (10-21) | 350 | 410 | 151 |
| TOTAL | 712 | 32.15  (17-80) | 13.00  (4-21) | 357 | 500 | 215 |

Table 3 iTaukei and North American participants by whether asked about God or not.

|  | Participants | Age | Education | Sex |
| --- | --- | --- | --- | --- |
|  | N | Mean  (min.- max.) | Mean  (min. – max.) | N Women |
| Yasawa | 91 | 44.39  (19-80) | 9.14  (5-15) | 30 |
| Indo-Fiji | 215 | 38.43  (17-76) | 10.54  (0-18) | 115 |
| US & Canada | 409 | 26.84  (17-72) | 13.64  (10-21) | 268 |
| TOTAL | 715 | 32.46  (17-80) | 12.16  (0-21) | 413 |

Table 4 Total sample participant numbers with demographics by sample

# Detailed Study 2 Procedure

For those who were also asked about God’s judgments, each judgment question was also followed by the same question asking what God would think about the actions in the vignette (e.g. “Would God think [perpetrator] should be rewarded or punished?”).

Our primed data in the third set of analyses were collected across 4-5 sessions, each lasting approx. 20 minutes and separated by approx. 24 hours. Participants answered one primed vignette per session, encompassing one of each intention condition (see Table 2) and one of each prime for each supernatural agent. Participants later answered questions about God, humans, and local ancestor spirits’ (Local gods) mental capacities in a fifth session. All materials were presented in counterbalanced order. Positive (desirable/ reward-worthy) and negative (undesirable/ punishment-worthy) thoughts or actions were presented as separate questions in counterbalanced order to balance out valence of question. Participants answered a Thought prime and an Action prime for both the Christian God and local ancestor spirits (4 total) in counterbalanced order (see McNamara et al., 2019, for further details).

In our first analyses, we focus on participants’ own judgments of how much reward or punishment the action deserved, as measured with the following questions:

Good/Bad: “How good or bad was what [perpetrator] did?”

Reward/ Punish: “In your opinion, do you think [perpetrator] should be rewarded or punished?”

We use intent condition (see Table 2), society, and whether or not participants were asked about God as our primary predictors of own punish/ reward judgments.

For our second and third set of analyses, we focus on participants’ expectations about God’s views of the relevant actions, i.e. how deserving of reward and punishment God will judge these actions. This was measured with the following question:

- Reward/ Punish: “Would God think (perpetrator) should be rewarded or punished?”)

We examine whether societies differ in how intentional they perceived the action to be and how bad they perceive the outcome to be for the victim by focusing our analysis on two questions:

- Intent = Purpose/Accident: “Did (perpetrator) do (action) on purpose or by accident?”
- Outcome = Positive/Negative: “How positively or negatively was (victim) affected?”

Our third set of analyses introduces a salience manipulation to induce participants to think about thoughts (Thought Prime) or think about actions (Action Prime) before considering each vignette. Primes are couched within questions similar to those in study 1: what do supernatural agents want and not want people to think or do? (See online supplement for questions.) We analyze the expectations of divine punishment as a function of priming condition and participants’ perception of how positive or negative the outcome was for the victim.

# Thought/ Action Prime Prompts

The Thought Prime asks participants to list up to 5 examples

- Thoughts God or the local ancestor spirits (Local gods) would or would not want them to think
- Whether that divine agent can reward or punish them for these thoughts
- (if they answered yes to possible reward or punishment) What kinds of rewards or punishments they might receive.

The Action Prime used the same wording but asked about what

- Actions God or the local ancestor spirits (Local gods) would or would not want them to do
- Whether that divine agent can reward or punish them for these actions
- (If yes to reward or punishment) what those punishments or rewards might be.

# Study 2 Full Regression Table

| Analysis 1 | | | | Analysis 2 | Analysis 3 | |  |  |
| --- | --- | --- | --- | --- | --- | --- | --- | --- |
|  | | S.Good_Bad  *b* [.95CI] | S.Reward_Punish  *b* [.95CI] | Expected God’s Punishment  *b* [.95CI] | Christian God  Punishment  *b* [.95CI] | *Kalou-vu*  Punishment  *b* [.95CI] |  |  |
| Intercept = Yasawa, Women, Accidents, Poison, Asked about God | | -1.43^***^  [-1.67, -1.20] | -1.40^***^  [-1.59, -1.21] | -- | -- | -- |  |  |
| Intercept = Yasawa, Women, Accidents, Poison | | -- | -- | 2.66^***^  [2.46, 2.86] | -- | -- |  |  |
| Intercept = Women, Action Prime, Accidents, Poison | |  |  |  | 2.20^***^  [1.38, 3.03] | 2.36^***^  [1.15, 3.58] |  |  |
| Slope of Perceived Actor Intent | | -- | -- | 0.03  [-0.05, 0.11] | 0.02  [-0.11, 0.15] | 0.16  [-0.04, 0.37] |  |  |
| Slope of Perceived Victim Outcome | | -- | -- | 0.17^***^  [0.08, 0.27] | 0.41^***^  [0.27, 0.55] | 0.14  [-0.06, 0.34] |  |  |
| Action vs. Thought Prime | |  |  |  | 0.30^*^  [0.05, 0.55] | 0.20  [-0.12, 0.53] |  |  |
| Yasawa vs. North American | | 0.54^***^  [0.37, 0.72] | 0.88^***^  [0.74, 1.02] | -0.50^***^  [-0.62, -0.37] | -- | -- |  |  |
| Yasawa vs. Indo-Fijian | | -- | -- | -0.39^***^  [-0.51, -0.27] | -- | -- |  |  |
| Accidents vs. Failed Attempts | | 0.40^***^  [0.25, 0.55] | 0.25^***^  [0.13, 0.37] | 0.25^***^  [0.15, 0.35] | 1.16^***^  [0.72, 1.61] | 0.73^*^  [0.07, 1.39] |  |  |
| Accidents vs. Intentional | | -0.30^***^  [-0.42, -0.17] | -0.44^***^  [-0.54, -0.34] | 0.37^***^  [0.28, 0.46] | 1.49^***^  [1.02, 1.97] | 0.87^*^  [0.04, 1.70] |  |  |
| Accidents vs. No Violation | | 1.10^***^  [0.95, 1.26] | 0.62^***^  [0.50, 0.75] | -0.60^***^  [-0.70, -0.50] | -0.75^**^  [-1.22, -0.28] | -1.02^**^  [-1.71, -0.32] |  |  |
| Asked about God vs. Not | | 0.15  [-0.09, 0.40] | 0.23^*^  [0.03, 0.43] | -- |  |  |  |  |
| Poison vs. Harm | | 0.42^***^  [0.37, 0.48] | 0.37^***^  [0.32, 0.42] | -0.20^***^  [-0.27, -0.12] |  |  |  |  |
| Poison vs. Theft | | 0.56^***^  [0.50, 0.62] | 0.36^***^  [0.31, 0.41] | -0.18^***^  [-0.25, -0.10] | -0.31  [-0.68, 0.07] | -0.36  [-0.86, 0.15] |  |  |
| Poison vs. Failed Cooperation | | 0.97^***^  [0.87, 1.07] | 0.70^***^  [0.61, 0.78] | -- | -0.59^**^  [-0.94, -0.24] | -0.67^*^  [-1.20, -0.14] |  |  |
| Poison vs. Food Taboo | | 0.96^***^  [0.90, 1.01] | 0.63^***^  [0.58, 0.68] | -0.25^***^  [-0.33, -0.18] |  |  |  |  |
| Poison vs. Social Taboo | | 0.64^***^  [0.54, 0.74] | 0.44^***^  [0.35, 0.52] | -- | 0.22  [-0.13, 0.58] | -0.01  [-0.50, 0.48] |  |  |
| Years Formal Education | | -0.001  [-0.02, 0.01] | -0.002  [-0.01, 0.01] | 0.02^**^  [0.01, 0.03] | -0.01  [-0.07, 0.05] | 0.02  [-0.06, 0.10] |  |  |
| Women vs. Men | | 0.03  [-0.03, 0.10] | -0.03  [-0.09, 0.02] | 0.08^**^  [0.02, 0.14] | 0.05  [-0.21, 0.30] | 0.08  [-0.25, 0.41] |  |  |
| Age | | 0.0003  [-0.003, 0.003] | 0.002^†^  [-0.0001, 0.004] | 0.001  [-0.001, 0.004] | -0.002  [-0.01, 0.01] | -0.005  [-0.02, 0.01] |  |  |
| Yasawa vs. North American for Accidents vs. Failed Attempts | | -1.02^***^  [-1.20, -0.83] | -0.84^***^  [-0.99, -0.69] | -- | -- | -- |  |  |
| Yasawa vs. North Americans for Accidents vs. Intentional | | -0.36^***^  [-0.51, -0.22] | 0.01  [-0.10, 0.13] | -- | -- | -- |  |  |
| Yasawa vs. North Americans for Accidents vs. No Violations | | -0.53^***^  [-0.71, -0.34] | -0.42^***^  [-0.57, -0.26] | -- | -- | -- |  |  |
| Yasawa vs. North American and Asked about God vs. Not | | -0.37^**^  [-0.64, -0.10] | -0.39^***^  [-0.61, -0.17] | -- | -- | -- |  |  |
| Asked about God vs. Not for Accidents vs. Failed Attempts | | -0.40^**^  [-0.66, -0.13] | -0.30^**^  [-0.52, -0.09] | -- | -- | -- |  |  |
| Asked about God vs. Not for Accidents vs. Intentional | | 0.04  [-0.21, 0.29] | 0.22^*^  [0.02, 0.43] | -- | -- | -- |  |  |
| Asked about God vs. Not for Accidents vs. No Violations | | -0.66^***^  [-0.93, -0.40] | -0.31^**^  [-0.52, -0.10] | -- | -- | -- |  |  |
| Yasawa vs. North American and Asked about God vs. Not for Accidents vs. Failed Attempts | | 0.71^***^  [0.40, 1.01] | 0.66^***^  [0.41, 0.91] | -- | -- | -- |  |  |
| Yasawa vs. North Americans and Asked about God vs. Not for Accidents vs. Intentional | | -0.09  [-0.37, 0.19] | -0.15  [-0.38, 0.08] | -- | -- | -- |  |  |
| Yasawa vs. North Americans and Asked about God vs. Not for Accidents vs. No Violations | | 1.27^***^  [0.96, 1.57] | 0.62^***^  [0.37, 0.86] | -- | -- | -- |  |  |
| Perceived Actor Intent x Perceived Victim Outcome | | -- | -- | 0.07^†^  [-0.01, 0.15] | -- | -- |  |  |
| Perceived Actor Intent in Yasawa vs. Indo-Fijian | | -- | -- | 0.07^†^  [-0.01, 0.16] | -- | -- |  |  |
| Perceived Actor Intent in Yasawa vs. North American | | -- | -- | 0.07  [-0.01, 0.15] | -- | -- |  |  |
| Perceived Victim Outcome in Yasawa vs. Indo-Fijian | | -- | -- | -0.10^†^  [-0.21, 0.002] | -- | -- |  |  |
| Perceived Victim Outcome in Yasawa vs. North American | | -- | -- | -0.16^**^  [-0.26, -0.05] | -- | -- |  |  |
| Perceived Actor Intent x Perceived Victim Outcome in Yasawa vs. Indo-Fijian | | -- | -- | -0.10^*^  [-0.18, -0.01] | -- | -- |  |  |
| Perceived Actor Intent x Perceived Victim Outcome in Yasawa vs. North American | | -- | -- | -0.03  [-0.12, 0.06] | -- | -- |  |  |
| Perceived Actor Intent x Perceived Victim Outcome | | -- | -- | -- | -0.01  [-0.08, 0.07] | 0.02  [-0.08, 0.12] |  |  |
| Perceived Actor Intent in Action vs. Thought Prime | | -- | -- | -- | -0.04  [-0.20, 0.12] | 0.01  [-0.19, 0.22] |  |  |
| Perceived Victim Outcome in Action vs. Thought Prime | | -- | -- | -- | -0.27^**^  [-0.44, -0.10] | 0.13  [-0.10, 0.36] |  |  |
| Perceived Actor Intent x Perceived Victim Outcome  in Action vs. Thought Prime | | -- | -- | -- | 0.04  [-0.06, 0.14] | 0.03  [-0.09, 0.16] |  |  |
|  | | | |  |  |  |  |  |
| Random effects | Variance [SD] Corr | | Variance [SD] Corr | Variance [SD] Corr | Variance [SD] | Variance [SD] |  |  |
| IID [Intercept] | 0.03 [0.16] | | 0.02 [0.14] | 0.05 [0.22] | 0.00 [0.00] | 0.00 [0.00] |  |  |
| Slope [Sample] | 0.18 [0.43] -0.78 | | 0.12 [0.34] -0.78 |  |  |  |  |  |
| Slope (Yas vs. Indo Fiji) |  | |  | 0.13 [0.36] -0.81 |  |  |  |  |
| Slope (Yas vs. North Am) |  | |  | 0.03 [0.18] -0.96 0.86 |  |  |  |  |
| Residual | 0.55 [0.74] | | 0.36 [0.6] | 0.48 [0.69] | 0.51 [0.72] | 0.90 [0.95] |  |  |
| Number of obs [n] | 5574 | | 5568 | 2730 | 144 | 144 |  |  |
| Groups [N] | 673 | | 673 | 679 | 72 | 72 |  |  |
|  | | | |  |  |  |  |  |
| Note: †p<0.1; *p<0.05; **p<0.01; ***p<0.001 | | | |  |  |  |  |  |

Table 3 Regression models for study 2 analyses (1) Permissibility and punish-worthiness ratings of accidents vs. other intent conditions by sample and for participants who were and were not asked about God’s judgments. (2) Expected God’s punishment or reward as predicted by perceived actor intent and severity of outcome across societies sampled (3) Expected Divine Punishment for Action vs. Thought primes asking about Christian God or Local ancestor spirits/ local gods on Actor Intent and Victim Outcome

1. Story contents for harm, theft, poison, and food taboo domain vignettes were adapted from materials used in (H. C. Barrett et al., 2016). See online supplement section S.M.1. for sample vignettes. [↑](#footnote-ref-1)
