# Supplementary material for: God's mind on morality
Source: Evol Hum Sci. 2021 Jan 11;3:e6. doi: 10.1017/ehs.2021.1 (PMC10427303; doi:10.1017/ehs.2021.1)
Supplement: Supplementary file 1 [file S2513843X21000013sup.zip › S2513843X21000013sup002.docx]

# Acknowledgements

The authors would like to thank Paula Tekei, Malakai Waqairadovu, Siteri Kalouniuca, and the 2012, 2013, and 2014 Yasawa Island research team for their tireless work in supporting data collection and processing for this study. We extend our deepest Vinaka sera vakalevu gratitude to Peceli Tavaga and family and the villages of Teci, Dalomo, Bukama, and Yasawa-i-rara in Yasawa, Fiji, for their hospitality and unwavering, enthusiastic participation.

# Author Contributions

McNamara: Contrubited study design to both studies, designed study materials for iTaukei and North American online data collection, coordinated data collection in Yasawa and North American samples, conducted the analyses in studies 1 and 2, and drafted the completed full article, and helped secure funding for all phases of the project.

Senanayake: Contributed to literature review on ethnographic research into Opacity of Mind, Cognitive Science of Religion, and assisted in compiling matterials for analyses in studies 1 and 2.

Willard: Contributed to study design in both studies, contributed literature review and cultural discussion for Indo-Fijian samples, designed study materials for Indo-Fijian participants, collected data among Indo-Fijian samples, contributed extensive edits toward final draft, and helped secure funding for data collection phases of the project.

Henrich: Contributed to study design in both studies, contributed literature reivew and theoretical background, contributed extensive edits toward final draft, and helped secure funding for data collection phases of the project.

# Financial Support

This research was supported by “The Emergence of Prosocial Religions” from the John Templeton Foundation (#40603), and the Cultural Evolution of Religion Research Consortium (CERC), funded by a Social Sciences and Humanities Research Council of Canada partnership grant (895–2011–1009) from the (J.H.); a University of British Columbia Hampton Fund Research Grant (R.A.M, A.K.W., J.H.); and by the Royal Society of New Zealand Marsden Fast-Start Grant “Unpacking Opacity of Mind” (R.A.M., Project ID: 17-VUW-022).

# Conflicts of Interest

Authors declare no conflicts of interest.

# Research Transparency and Reproducibility

All de-identified data and analysis codes can be found at the project OSF page: https://osf.io/mzky8/?view_only=0020266e0736419e84116487df3408b9
